# Does PSA Nadir + 2 ng/mL Always Indicate Biochemical Recurrence? A PSA Kinetics-Based Evaluation Following Carbon Ion Radiotherapy for Localized High-Risk Prostate Cancer

**DOI:** 10.3390/cancers17172867

**Published:** 2025-08-31

**Authors:** Satoshi Shima, Yosuke Takakusagi, Tatsuya Okuda, Hiroaki Koge, Kio Kano, Kohei Okada, Keisuke Tsuchida, Shohei Kawashiro, Nobutaka Mizoguchi, Daisaku Yoshida, Hiroyuki Katoh, Takashi Uno

**Affiliations:** 1 Department of Radiation Oncology, Kanagawa Cancer Center, Yokohama, Kanagawa, Japan; t-okuda@kcch.jp (T.O.); kooge.9m70m@kanagawa-pho.jp (H.K.); kanou.1210m@kanagawa-pho.jp (K.K.); okada.1n80o@kanagawa-pho.jp (K.O.); ketsuchi@kcch.jp (K.T.); mizoguchin@kcch.jp (N.M.); d.yoshida@kcch.jp (D.Y.); katoh37hiroyuki@gmail.com (H.K.); 2 Diagnostic Radiology and Radiation Oncology, Graduate School of Medicine, Chiba University, Chiba, Chiba, Japan; unotakas@faculty.chiba-u.jp; 3 Department of Radiation Oncology, Yokohama Sakae Kyosai Hospital, Yokohama, Kanagawa, Japan; 4 Medical Care Bureau, Yokohama, Kanagawa, Japan; sh00-kawashiro@city.yokohama.lg.jp

**Keywords:** prostate cancer, carbon ion radiotherapy, prostate-specific antigen kinetics, biochemical recurrence, Phoenix criteria, prostate-specific antigen bounce

## Abstract

**Background:** Biochemical recurrence after radiotherapy for prostate cancer is commonly defined by the Phoenix criteria (prostate-specific antigen [PSA] nadir + 2 ng/mL). However, some patients experience PSA elevation without clinical recurrence, which is known as PSA bounce. This study aimed to evaluate PSA kinetics after scanning-method carbon ion radiotherapy (CIRT) in patients with high-risk prostate cancer (HR-PCa) and to assess the clinical validity of the Phoenix criteria. **Methods:** We retrospectively analyzed 171 patients with HR-PCa who underwent CIRT and 2 years of androgen deprivation therapy. Patients were classified into three groups based on post-treatment PSA kinetics: non-recurrence, pseudo-recurrence (PR; PSA > 2 ng/mL followed by spontaneous decline without salvage therapy), and recurrence (R; PSA > 2 ng/mL with salvage therapy). PSA bounce was defined as a transient PSA increase > 0.4 ng/mL followed by spontaneous decline. Kaplan–Meier and receiver operating characteristic (ROC) analyses were used to evaluate biochemical relapse-free survival and determine the optimal PSA cutoff. **Results:** Among 171 patients, 18 (10.5%) met the Phoenix criteria (R+PR), of whom 6 (33.3%) experienced spontaneous PSA decline. The 5-year biochemical relapse-free survival rate was 90.0%. PSA bounce occurred in 33.9%. ROC analysis identified an optimal PSA cutoff of 1.91 ng/mL (area under the curve: 0.985), whereas the positive predictive value at the 2 ng/mL cutoff was as low as 61.1% due to the influence of PSA bounce. **Conclusions:** After CIRT, a PSA rise of >2 ng/mL does not always indicate HR-PCa recurrence and should be interpreted with caution to avoid overtreatment.

## Simple Summary

Biochemical recurrence after radiotherapy for prostate cancer is often defined using the Phoenix criteria (prostate-specific antigen [PSA] nadir + 2 ng/mL). However, some patients experience a temporary PSA increase followed by spontaneous decline, known as PSA bounce, which may lead to overdiagnosis. Data on PSA kinetics following carbon ion radiotherapy (CIRT) in high-risk prostate cancer (HR-PCa) remain limited. This study analyzed 171 patients with HR-PCa treated with CIRT. Eighteen patients (10.5%) met the Phoenix criteria, but six (33.3%) showed spontaneous PSA decline. PSA bounce occurred in 33.9%. The optimal PSA cutoff for true recurrence was 1.91 ng/mL, but the standard 2.0 ng/mL cutoff had a low positive predictive value (61.1%) due to PSA bounce. These findings suggest that not all rises in recurrent PSA beyond nadir + 2 ng/mL indicate recurrence. Careful monitoring of PSA kinetics is essential to avoid unnecessary treatment.

## 1. Introduction

Prostate cancer is the most commonly diagnosed cancer among men in Japan [1] and the fourth most common cancer worldwide [2]. Radiation therapy is a definitive treatment for localized prostate cancer, with three available modalities: external beam radiation therapy using X-rays (such as intensity-modulated radiation therapy [IMRT] and stereotactic body radiation therapy [SBRT]), brachytherapy, and particle therapy [3,4].

Carbon ion radiotherapy (CIRT), a type of particle therapy, provides high dose concentration due to the Bragg peak and low penumbra, potentially reducing radiation exposure to adjacent normal tissues compared with IMRT [5]. Additionally, CIRT induces more DNA double-strand breaks and has greater biological efficacy than X-ray or proton therapies [6,7].

Prostate-specific antigen (PSA) is a sensitive serum marker for prostate cancer [8]. Recurrence after radiation therapy is commonly assessed using the Phoenix criteria (PSA nadir + 2 ng/mL) [9]. However, transient PSA elevations, referred to as PSA bounce, can occur without clinical recurrence. Originally observed in brachytherapy [10], PSA bounce has also been observed in IMRT and CIRT [11]. While numerous studies have investigated PSA bounce following radiation therapies such as brachytherapy [12,13], IMRT [14], and SBRT [15], studies concerning CIRT remain exceptionally scarce. Accurate interpretation of PSA kinetics during follow-up is essential to avoid unnecessary salvage therapy.

Takakusagi et al. reported cases in the D’Amico intermediate-risk group wherein PSA levels spontaneously declined after exceeding 2 ng/mL without salvage therapy [16]. To our knowledge, no similar studies have focused on the D’Amico high-risk group. Therefore, this study aimed to (1) determine the proportion of patients with high-risk prostate cancer (HR-PCa) who experienced spontaneous PSA decline after CIRT, following fulfillment of the Phoenix criteria, and (2) assess the validity of those criteria for guiding salvage treatment initiation.

## 2. Materials and Methods

### 2.1. Patient Eligibility

In 2015, our institution began using a scanning method for CIRT. Prostate cancer cases were classified according to the D’Amico classification system [17]. From radiation therapy records, we identified 240 patients classified as high-risk among 389 patients with prostate cancer treated between 15 December 2015 and 31 November 2018. Eligible patients had a follow-up period of at least 54 months, received androgen deprivation therapy (ADT) for 12–36 months, and met the Phoenix criteria at the time of recurrence diagnosis and salvage therapy initiation. In CIRT for prostate cancer, the treatment protocol at our institution is as follows: No ADT is combined for low-risk patients; 6 months of ADT are combined for intermediate-risk patients; and 2 years of ADT are combined for high-risk patients. Patients whose treatment clearly deviated from this institutional treatment protocol were excluded from this study. The lower limit for the follow-up period was set at 54 months to allow a 6-month margin for data collection around the fifth year.

This study was approved by the Institutional Review Board of our institution, Kanagawa Cancer Center Ethics Review Committee (approval number: 2023Eki-124), and written informed consent was obtained from all participants. This study was conducted in accordance with the Declaration of Helsinki.

### 2.2. ADT

All patients received 2 years of ADT, consisting of combined androgen blockade with subcutaneous luteinizing hormone-releasing hormone (LH-RH) agonists and oral antiandrogens. Antiandrogens were discontinued if complications arose; however, LH-RH agonists were mandatory. A minimum of 3 months of ADT was required prior to CIRT.

### 2.3. CIRT

Detailed CIRT methods have been described by Takakusagi et al. [18]. Briefly, patients were immobilized in the supine position using a vacuum mattress (BlueBag; Elekta, Stockholm, Sweden) and a thermoplastic shell (Shellfitter; Kuraray, Tokyo, Japan). Computed tomography (CT) and magnetic resonance imaging (MRI) scans were acquired and fused using MIM Maestro software (version 6.6 and 7.3, MIM Software Inc., Cleveland, OH, USA) for radiation therapy planning. The clinical target volume (CTV) included the entire prostate and the base of the seminal vesicles. In cases of seminal vesicle invasion, the entire affected seminal vesicle was included. Margins of 4 mm posteriorly, 5 mm superiorly and inferiorly, and 10 mm laterally and anteriorly were added to the CTV to define the planning target volume (PTV), which was adjusted as needed to meet dose constraints for the small intestine and sigmoid colon.

The prescribed dose was 51.6 GyE in 12 fractions, calculated using the Monaco Carbon Scanning System (version 5.1 and 6.1, Elekta, Stockholm, Sweden). Treatment used two or four lateral beams delivered once daily, four times per week, alternating sides. No hydrogel spacers or gold fiducial markers were used. Laxatives and glycerin enemas were administered prior to irradiation as needed for bowel preparation. Patients hydrated prior to irradiation to ensure adequate bladder filling, verified using the Bladder Scan System (Sysmex, Kobe, Japan). X-ray imaging was performed using a diagnostic X-ray flat-panel detector imaging system (RADspeed, Shimadzu, Kyoto, Japan) immediately prior to treatment in both horizontal and vertical directions to confirm positioning. If rectal gas/stool or insufficient bladder filling interfered with targeting, treatment was paused, and enemas or hydration was repeated. In-room CT was performed at least once weekly to verify organ positioning [19].

### 2.4. Follow-Up

PSA levels were monitored every 3 months for the first 3 years, every 6 months until the fifth year, and annually thereafter. Biochemical recurrence was defined as a PSA increase ≥ 2 ng/mL above the nadir, according to the Phoenix criteria [9]. Patients with suspected recurrence underwent CT, MRI, and bone scintigraphy, and salvage ADT was initiated at the discretion of the urologist. Recurrence was defined by the initiation of salvage therapy, but patients continued follow-up regardless.

Patients were categorized into three groups based on PSA kinetics: non-recurrence (NR), pseudo-recurrence (PR), and recurrence (R). PR was defined as PSA elevation ≥ 2 ng/mL above the nadir that spontaneously decreased without salvage therapy. R was defined as PSA elevation ≥ 2 ng/mL above the nadir followed by salvage ADT. In accordance with previous studies, PSA bounce was defined as a transient PSA rise ≥ 0.4 ng/mL followed by spontaneous decline [16].

### 2.5. Data Analysis

Data collected on 1 June 2024 included age, clinical stage, Gleason score, ADT duration, radiotherapy dates, and PSA levels throughout follow-up. PSA trends were visualized using Microsoft 365 (Microsoft Corp., Redmond, WA, USA). Kaplan–Meier curve and receiver operating characteristic (ROC) curves were generated using Python (version 3.11.5) [20] with the libraries matplotlib (version 3.8.0) [21] and scikit-learn (version 1.3.0) [22]. In ROC analysis, negative cases were defined by the peak PSA level observed during follow-up in the NR and PR groups, whereas positive cases were defined by the PSA level observed immediately before the initiation of salvage ADT in the R group. The Youden index, calculated in Python, was used to determine the optimal cutoff for identifying biochemical recurrence.

### 2.6. Statistical Analysis

All statistical analyses were conducted using Python with the libraries Pandas (version 2.0.3) [23], NumPy (version 1.24.3) [24], and scipy.stats (SciPy version 1.11.1) [25]. Continuous variables were compared among the three groups using the Kruskal–Wallis test with Bonferroni correction for multiple comparisons, and pairwise comparisons were assessed using the Mann–Whitney U test. Categorical variables were analyzed using the chi-square test. Effect sizes were calculated using η^2^ for continuous variables and Cramer’s V for categorical variables. Statistical significance was set at *p* < 0.05.

## 3. Results

### 3.1. Patient Characteristics

**Figure 1:**
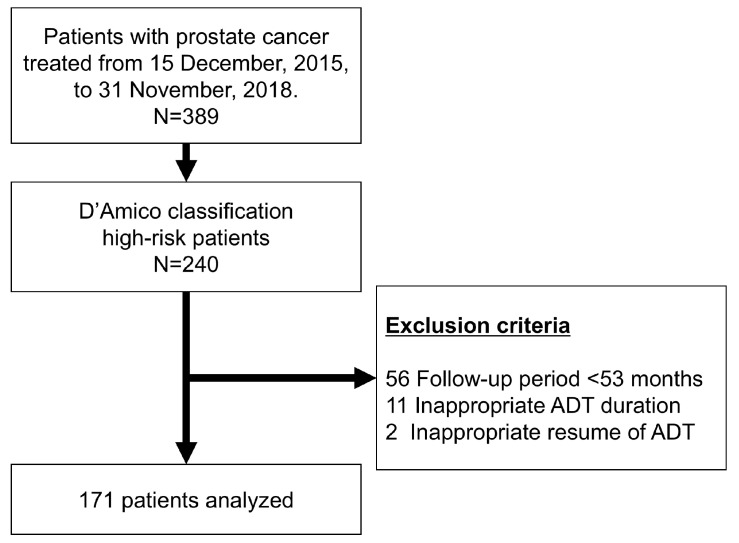
CONSORT diagram of this study. Among 389 patients who underwent CIRT between 15 December 2015, and 31 November 2018, 240 were classified as D’Amico high-risk. Of these, 69 were excluded. A total of 171 patients were included in the analysis. ADT, androgen deprivation therapy; CIRT, carbon ion radiotherapy.

A CONSORT diagram is shown in Figure 1. Based on the eligibility criteria, 56 patients with a follow-up period of <53 months, 11 patients with ADT duration of < 1 year or >3 years, and 2 patients in whom salvage ADT was clearly initiated at an inappropriate time (e.g., initiated when PSA exceeded 0.2 ng/mL without any imaging evaluation) were excluded. A total of 171 patients were included in the analysis. Their background characteristics are summarized in Table 1. The median follow-up duration was 69 months (range, 47–95 months), and the median age was 70 years (range, 47–84 years). According to the UICC 8th edition, clinical T stages were distributed as follows: T1c (n = 16), T2a (n = 38), T2b (n = 24), T2c (n = 49), T3a (n = 34), T3b (n = 10), and T4 (n = 0). PSA levels prior to prostate biopsy were ≤10 in 80 cases, 10 to 20 in 55, and ≥20 in 36. Gleason scores were 6 (n = 3), 7 (n = 31), 8 (n = 91), and 9 (n = 46). All patients received ADT for a median duration of 24 months (range, 12–36 months). Two patients died of other causes during follow-up.

### 3.2. Clinical Outcome

**Figure 2:**
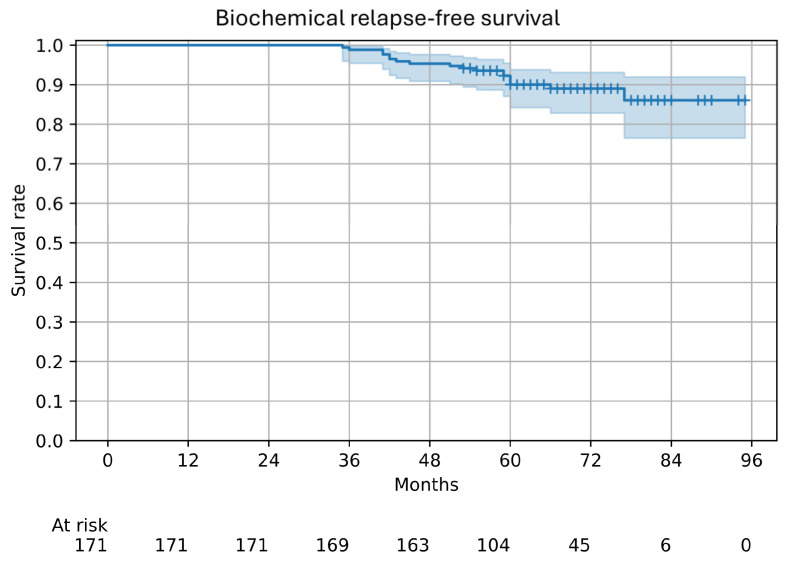
Kaplan–Meier curve for biochemical relapse-free survival. The 5-year biochemical relapse-free survival rate was 90.0% (95% confidence interval [CI], 84.2–93.8). Censoring is indicated by “+”, and the 95% CI is shown as shaded bands above and below the solid line.

Kaplan–Meier curves for biochemical recurrence are shown in Figure 2. The 5-year biochemical relapse-free survival rate was 90.0% (95% confidence interval [CI], 84.2–93.8), including the PR group. Details of the 12 patients in the R group are summarized in Table 2. Recurrence sites were identified as the prostate (n = 3), lymph nodes (n = 2), and bone (n = 1). Based on evaluations with CT, MRI, and bone scintigraphy; none of the patients underwent prostate-specific membrane antigen positron emission tomography (PSMA-PET) imaging, which remains available at a very limited number of institutions in Japan. The remaining six patients had no identifiable lesions based on imaging, but recurrence was diagnosed based on sustained PSA elevation and initiation of salvage therapy. Among the three patients with local recurrence, the involved sites were the seminal vesicles plus the left anterior lobe of the prostate, left anterior plus right posterior lobe, and both posterior lobes.

### 3.3. Comparison of Patient Backgrounds

Based on PSA progression, patients were classified into the NR (n = 153), PR (n = 6), and R (n = 12) groups. Patient characteristics are summarized in Table 3. Significant differences among the three groups were observed in T stage (*p* = 0.043, Cramer’s V = 0.234), Gleason score (*p* = 0.002, Cramer’s V = 0.25), and PSA nadir (*p* = 0.011, η^2^ = 0.042). T stage and Gleason score differed significantly between the NR and R groups (*p* = 0.021 and 0.031, respectively). Recurrence was not observed in patients with Gleason scores of 6 or 7, whereas it was observed in 4.4% and 17.4% of patients with scores of 8 and 9, respectively. Although PSA nadir differed significantly among the three groups, post hoc analysis showed no significant pairwise differences after Bonferroni correction.

### 3.4. PSA Kinetics

**Figure 3:**
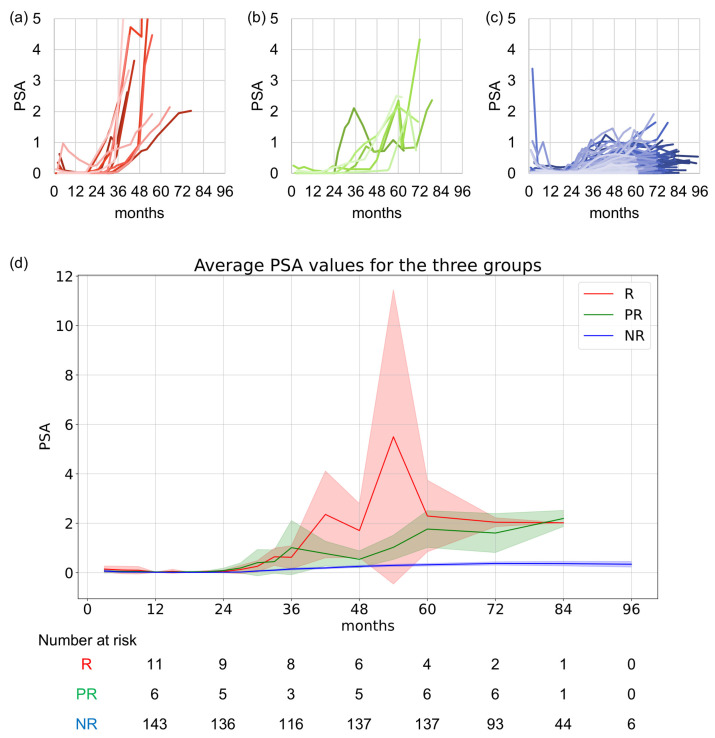
PSA kinetics in the three groups. (**a**) PSA trend in the recurrence (R) group, which met the Phoenix criteria and received salvage ADT. (**b**) PSA trend in the pseudorecurrence (PR) group, which met Phoenix criteria but experienced spontaneous PSA decline without treatment. (**c**) PSA trend in the non-recurrence (NR) group, which did not meet the Phoenix criteria. Cases are color-coded in varying shades to improve visual distinction. (**d**) Average PSA trends at 3-, 6-, and 12-month follow-ups. Red indicates R group. Green indicates PR group. Blue indicates NR group. The solid line shows the average PSA level, and the shaded areas indicate 95% confidence intervals. Significant differences were observed between the NR and PR groups and between the NR and R groups. No significant differences were observed between the PR and R groups at any time point. PSA, prostate-specific antigen; ADT, androgen deprivation therapy.

PSA trends are shown in Figure 3. In the R group, post-salvage ADT data were omitted for clarity (Figure 3a). Figure 3d summarizes the average PSA kinetics at 3-, 6-, and 12-month follow-ups. Significant differences among the three groups were observed at 21, 27, 36, 42, 48, 54, 60, and 72 months (*p* = 0.033, 0.008, 0.001, 0.000, 0.000, 0.000, 0.000, and 0.000, respectively). Significant differences were observed between the NR and PR groups at 27, 30, 36, 42, 54, 60, 72, and 84 months (*p* = 0.005, 0.034, 0.006, 0.012, 0.003, 0.001, 0.000, and 0.044, respectively). Similarly, significant differences were observed between the NR and R groups at 21, 36, 42, 48, 54, 60, 72, and 84 months (*p* = 0.048, 0.004, 0.000, 0.000, 0.000, 0.001, 0.000, and 0.044, respectively). No significant differences were observed between the PR and R groups at any time point.

### 3.5. PSA Bounce

PSA bounce occurred in 48 patients (31.3%) in the NR group. All six patients in the PR group met the criteria for PSA bounce, as PSA levels spontaneously decreased after exceeding 0.4 ng/mL. Overall, PSA bounce was observed in 54 patients (33.9%). As shown in Table 4, patients with PSA bounce were significantly younger than patients with no bounce (*p* = 0.004, r = −0.23). No significant differences were observed in T stage, Gleason score, pretreatment PSA, PSA nadir, or ADT duration.

### 3.6. PSA Cutoff Value

**Figure 4:**
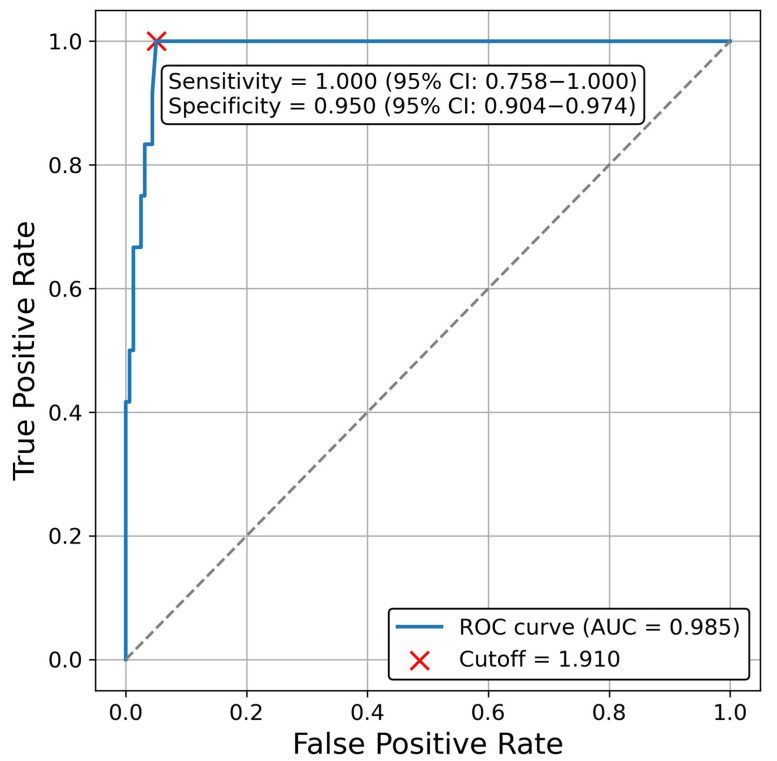
ROC curve and optimal cutoff value. ROC analysis identified an optimal PSA cutoff of 1.91 ng/mL with an AUC of 0.985, sensitivity of 100%, and specificity of 95%. This value was similar to that of the Phoenix criteria. ROC, receiver operating characteristic; PSA, prostate-specific antigen; AUC, area under the curve.

The ROC curve is shown in Figure 4. The optimal cutoff value was 1.91 ng/mL, with an area under the curve of 0.985. At this cutoff, sensitivity and specificity were 100% (95% CI, 75.8–100) and 95.0% (95% CI, 90.4–97.4), respectively. Evaluation results for cutoff values of 1 to 6 ng/mL are shown in Table 5. When the cutoff values were set at 1, 2, 3, 4, and 5 ng/mL, the sensitivities were 100%, 91.7%, 58.3%, 41.7%, and 25%, respectively; the specificities were 87.4%, 95.6%, 98.7%, 99.3%, and 100%, respectively; and the positive predictive values for recurrence were 37.5%, 61.1%, 77.8%, 83.3%, and 100%, respectively.

## 4. Discussion

This study evaluated treatment outcomes and PSA kinetics in patients with HR-PCa undergoing CIRT, classified according to the D’Amico criteria. Previous reports on X-ray therapy reported 5-year recurrence-free survival rates of 89% and 68% in Cambridge prognostic groups 4 and 5, respectively [26]. In the ASCENDE-RT trial, Morris et al. reported 5-year biochemical progression-free survival rates of 84.9% and 89.7% in intermediate-risk and high-risk patients who received whole pelvic irradiation plus either external beam radiation therapy or low-dose rate boost, respectively [27]. For brachytherapy alone, Yoshioka et al. reported a 5-year biochemical recurrence-free survival rate of 81% in HR-PCa cases [28]. For proton therapy, Takagi et al. reported 5-year disease-free survival rates of 90%, 88%, and 76% in cases classified as unfavorable intermediate, high, and very high risk, respectively [29]. With CIRT, Nomiya et al. reported a 5-year disease-free survival rate of 92% in D’Amico high-risk patients [30], consistent with the current study.

Analysis of patient background characteristics revealed significant differences in Gleason score and T stage among the three groups, suggesting their potential influence on post-treatment PSA elevation. Kasuya et al. identified T3b, Gleason score 9 or 10, and >75% positive biopsy cores as predictors of prostate cancer-specific mortality after CIRT [31]. On the other hand, D’Amico et al. identified high PSA levels, Gleason scores 8 to 10, advanced T stage, and short ADT duration as recurrence risk factors after X-ray therapy [32]. Additionally, Proust-Lima et al. found that long-term PSA elevation after radiation therapy was associated with pretreatment PSA level, T stage, and Gleason score [33]. In our study, pretreatment PSA was not significantly associated with recurrence, possibly reflecting differences in treatment modalities.

The PR group was defined as cases in which PSA spontaneously decreased after meeting the Phoenix criteria. These cases are often misinterpreted as true recurrence in clinical practice, potentially leading to overtreatment. In a prior study of patients with intermediate risk classified according to the D’Amico classification, Takakusagi et al. reported PSA failure in 9.4%, with 87.5% showing spontaneous PSA decline, and that the PSA bounce rate at a cutoff of 0.4 ng/mL was 45.9% [16]. In their analysis, younger age and lower T stage were significant predictors of PSA bounce. In our study, which targeted high-risk patients classified according to the D’Amico classification, 18 (10.5%) experienced PSA failure, with only 6 (33.3%) exhibiting spontaneous decline. PSA bounce occurred in 31.3% of the NR group and 33.9% overall (NR + PR groups), lower than in intermediate-risk patients, and younger age was also associated with PSA bounce. The lower incidence of PSA bounce in high-risk patients compared with intermediate-risk patients may be attributable to differences in ADT duration (6 months vs. 2 years), which could have influenced PSA kinetics. In a study by Nam et al., patients who continued ADT for >18 months had significantly lower rates of testosterone recovery (27.5% vs. 74.6%) and longer median recovery times (6.8 months vs. 9.7 months) than those who continued ADT for <18 months [34]. Taken together, these results suggest that spontaneous PSA declines are less common in high-risk patients, emphasizing the importance of recurrence-focused management during follow-up.

ROC analysis identified an optimal PSA cutoff of 1.91 ng/mL for predicting recurrence, close to the Phoenix criteria (2 ng/mL) that is commonly used in clinical practice. However, the positive predictive value at 2 ng/mL was reduced under the influence of PSA bounce. Ma et al. reported a 30.2% false positive rate using 2 ng/mL as the cutoff [35]. A meta-analysis by Darwis et al. reported that CIRT was more prone to PSA bounce than other modalities [36]. According to that meta-analysis, the incidence of PSA bounce was 34% with low-dose-rate brachytherapy, 36% with high-dose-rate brachytherapy, 22% with external beam radiation therapy, 28% with SBRT, and 56% with CIRT. Considering that PSA bounce occurs relatively frequently after CIRT and that salvage ADT often entails long-term treatment and potential adverse effects, especially when nonpharmacological salvage options are limited, it may not be appropriate to initiate salvage therapy solely on the basis of PSA exceeding 2 ng/mL. Patients should instead be closely monitored. In our cohort, PSA bounce was less frequent in older patients.

This study had some limitations. First, it was conducted at a single institution with a retrospective design, introducing potential bias. Second, the absence of a standardized protocol for defining recurrence and initiating salvage therapy allowed for physician discretion, which could have led to inconsistencies in clinical judgment and reduced the reproducibility of our findings. Third, classification into the PR and R groups was based on whether salvage therapy was initiated, which was subject to physician preferences, potentially introducing classification bias. To further improve specificity, next-generation imaging techniques such as PSMA-PET are warranted. Furthermore, the small number of PR and R cases limit the robustness of the subgroup analysis. Given the slow progression of prostate cancer and reports supporting > 10-year follow-up [4], longer follow-up and larger sample sizes are warranted to validate our findings.

## 5. Conclusions

This study investigated PSA kinetics after CIRT in patients with HR-PCa. While some patients showed spontaneous PSA declines after exceeding 2 ng/mL, others showed sustained increases, indicating recurrence. PSA elevations ≥ 2 ng/mL do not always indicate true recurrence, highlighting the need for careful monitoring to avoid unnecessary salvage therapy. To minimize overtreatment, further prospective studies, particularly multi-institutional validations, are needed to establish more accurate PSA-based criteria for recurrence after CIRT.

## Figures and Tables

**Table 1:** Patient characteristics (n = 171).

Characteristics				n (%)
	Follow-up duration, months, median (range)			69 (47–95)
	Age, years, median (range)			70 (47–84)
	D’Amico classification			
		High		171 (100%)
	T stage			
		1c		16 (9.4%)
		2a		38 (22.2%)
		2b		24 (14.0%)
		2c		49 (28.7%)
		3a		34 (19.9%)
		3b		10 (5.8%)
		4		0 (0%)
	Pretreatment PSA, ng/mL, median (range)			10.2 (3.37–187)
		≤10		80 (46.8%)
		10 ≤ 20		55 (32.2%)
		<20		36 (21.0%)
	Gleason Score			
		6		3 (1.8%)
		7		31 (18.1%)
		8		91 (53.2%)
		9		46 (26.9%)
	ADT			
		neoadjuvant		171 (100%)
		adjuvant		171 (100%)
		duration, month, median (range)		24 (12–36)

PSA, prostate-specific antigen; ADT, androgen deprivation therapy.

**Table 2:** Details of patients in the recurrent group.

No.	Age	T Stage	Pretreatment PSA (ng/mL)	GS	ADT Duration (Months)	Time to Recurrence (Months)	Clinical Recurrence	Site
1	68	T3a	34	4 + 4 = 8	29	41	No	-
2	66	T3a	13.49	4 + 4 = 8	27	77	No	-
3	63	T3a	11.257	4 + 5 = 9	26	45	Yes	Lumber bone
4	69	T2b	7.5	4 + 5 = 9	24	36	Yes	Left external iliac lymph node
5	68	T3b	10.4	4 + 5 = 9	18	53	No	-
6	72	T3a	23.97	4 + 5 = 9	24	54	Yes	Prostate, left obturator lymph node
7	65	T3b	10.1	5 + 4 = 9	25	55	No	-
8	73	T2c	7.36	4 + 5 = 9	15	42	Yes	Multiple pelvic lymph nodes
9	70	T2a	20.244	4 + 4 = 8	24	65	Yes	Prostate
10	69	T2a	10.2	4 + 4 = 8	27	55	No	-
11	68	T3b	35	4 + 5 = 9	22	42	Yes	Prostate
12	75	T3a	48.65	4 + 5 = 9	22	41	No	-

PSA, prostate-specific antigen; GS, Gleason Score; ADT, androgen deprivation therapy.

**Table 3:** Detailed patient characteristics by recurrence status.

Characteristics				n (%)							
	Group			Non-Recurrence (n = 153)	Pseudo-Recurrence (n = 6)	Recurrence (n = 12)	*p*-Value ^1^	Effect Size	NR vs. PR ^2^	NR vs. R ^2^	PR ^2^ vs. R ^2^
	Follow-up duration, months, median (range)			69 (53–95)	71 (62–79)	63 (36–77)	0.218	η^2^ = 0.006	-	-	-
	Age, years, median (range)			70 (47–84)	65 (55–70)	68 (63–75)	0.07	η^2^ = 0.020	-	-	-
	T stage						0.043	Cramer’s V = 0.234	1	0.021	1
		1c		16 (10.5%)	0 (0%)	0 (0%)					
		2a		35 (22.9%)	1 (16.7%)	2 (16.7%)					
		2b		23 (15.0%)	0 (0%)	1 (8.3%)					
		2c		46 (30.1%)	2 (33.3%)	1 (8.3%)					
		3a		27 (17.6%)	2 (33.3%)	5 (41.6%)					
		3b		6 (3.9%)	1 (16.7%)	3 (25%)					
		4		0 (0%)	0 (0%)	0 (0%)					
	Pretreatment PSA, ng/mL, median (range)			10.09 (3.37–187)	26.81 (4.95–108.64)	12.37 (7.36–48.65)	0.07	η^2^ = 0.020	-	-	-
		≤10		76 (49.7%)	2 (33.3%)	2 (16.7%)					
		10 ≤ 20		50 (32.7%)	0 (0%)	5 (41.7%)					
		>20		27 (17.6%)	4 (66.7%)	5 (41.7%)					
	Gleason score						0.002	Cramer’s V = 0.25	0.064	0.031	0.197
		6		2 (1.3%)	1 (16.7%)	0 (0%)					
		7		29 (19.0%)	2 (33.3%)	0 (0%)					
		8		86 (56.2%)	1 (16.7%)	4 (33.3%)					
		9		36 (23.5%)	2 (33.3%)	8 (66.7%)					
	ADT										
		neoadjuvant		153 (100%)	6 (100%)	12 (100%)	-				
		adjuvant		153 (100%)	6 (100%)	12 (100%)	-				
	duration, month, median (range)			24 (12–36)	24 (22–27)	24 (15–29)	0.912	η^2^ = −0.010	-	-	-
	PSA nadir, ng/mL, median (range)			0.008 (0–0.1)	0.011 (0.001–0.06)	0.008 (0.003–0.17)	0.011	η^2^ = 0.042	0.089	0.089	1

^1^ Overall comparisons among the three groups were performed using the Kruskal–Wallis test for continuous variables and the chi-square test for categorical variables. ^2^ Post hoc pairwise comparisons were performed using the Mann–Whitney U test with Bonferroni correction for continuous variables and the chi-square test for categorical variables. NR, non-recurrence; PR, pseudo-recurrence; R, recurrence; PSA, prostate-specific antigen; ADT, androgen deprivation therapy.

**Table 4:** Characteristics of no-bounce and bounce groups.

		No-Bounce (n = 105)	Bounce (n = 48)	*p*-Value ^1^	Effect Size
Age, years, median (range)		71 (57–84)	68 (47–79)	0.004	r = −0.23
T stage				0.143	Cramer’s V = 0.23
	1c	7 (6.7%)	9 (18.8%)		
	2a	26 (24.8%)	9 (18.8%)		
	2b	15 (14.3%)	8 (16.7%)		
	2c	33 (31.4%)	13 (27.1%)		
	3a	18 (17.1%)	9 (18.8%)		
	3b	6 (5.7%)	0 (0%)		
	4	0 (0%)	0 (0%)		
Pretreatment PSA, ng/mL, median (range)		9.2 (3.37–187)	10.675 (4.02–84.3)	0.665	r = 0.035
	≤10	56 (53.3%)	20 (41.7%)		
	10 ≤ 20	30 (28.6%)	20 (41.7%)		
	<20	19 (18.1%)	8 (16.7%)		
PSA nadir, ng/mL, median (range)		0.008 (0–0.1)	0.008 (0–0.062)	0.345	r = 0.076
Gleason Score				0.182	Cramer’s V = 0.18
	6	2 (1.9%)	0 (0%)		
	7	17 (16.2%)	12 (25%)		
	8	57 (54.3%)	29 (60.4%)		
	9	29 (27.6%)	7 (14.6%)		
ADT duration, months, median (range)		25 (13–36)	24 (12–35)	0.256	r = −0.092

^1^ Overall comparisons were performed using the Mann–Whitney U test for continuous variables and the chi-square test for categorical variables. PSA, prostate-specific antigen; ADT, androgen deprivation therapy.

**Table 5:** PSA cutoff value and positive predictive value for recurrence.

PSA Cutoff Value (ng/mL)		1	2	3	4	5
	Positive predictive value (%)	37.5	61.1	77.8	83.3	100
	Sensitivity (%)	100	91.7	58.3	41.7	25
	Specificity (%)	87.4	95.6	98.7	99.3	100

PSA, prostate-specific antigen.

## Data Availability

The data presented in this study is available upon request from the corresponding author.

## Abbreviations

The following abbreviations are used in this manuscript:

CIRT	carbon ion radiotherapy
PSA	prostate-specific antigen
ADT	androgen deprivation therapy
LH-RH	luteinizing hormone-releasing hormone
CT	computed tomography
MRI	magnetic resonance imaging
CTV	clinical target volume
PTV	planning target volume
HR-PCa	high-risk prostate cancer
NR	non-recurrence
PR	pseudorecurrence
R	recurrence
AUC	area under the curve
ROC	receiver operating characteristic
